# Sound can suppress visual perception

**DOI:** 10.1038/srep10483

**Published:** 2015-05-29

**Authors:** Souta Hidaka, Masakazu Ide

**Affiliations:** 1 Department of Psychology, Rikkyo University, 1-2-26, Kitano, Niiza-shi, Saitama, 352-8558 Japan.; 2 Developmental Disorders Section, Department of Rehabilitation for Brain Functions, Research Institute of National Rehabilitation Center for Persons with Disabilities, 4-1, Namiki, Tokorozawa-shi, Saitama, 359-8555 Japan

## Abstract

In a single modality, the percept of an input (e.g., voices of neighbors) is often suppressed by another (e.g., the sound of a car horn nearby) due to close interactions of neural responses to these inputs. Recent studies have also suggested that close interactions of neural responses could occur even across sensory modalities, especially for audio-visual interactions. However, direct behavioral evidence regarding the audio-visual perceptual suppression effect has not been reported in a study with humans. Here, we investigated whether sound could have a suppressive effect on visual perception. We found that white noise bursts presented through headphones degraded visual orientation discrimination performance. This auditory suppression effect on visual perception frequently occurred when these inputs were presented in a spatially and temporally consistent manner. These results indicate that the perceptual suppression effect could occur across auditory and visual modalities based on close and direct neural interactions among those sensory inputs.

When taking a walk in a street, we may see many objects (e.g., peoples, trees, and cars) and hear a variety of sounds (e.g., talking voices, wind blowing, and car horns) simultaneously. Our perceptual systems in the brain automatically and efficiently integrate these inputs, not only within a single modality (e.g., colors and shapes in vision), but also across sensory modalities (e.g., peoples’ appearance and their voices). This process contributes to establishing coherent and robust percepts regarding our surrounding environment^1^.

The processes of integration are generally considered to mean the conjunction and/or summation of multiple pieces of information. However, sensory information is often perceptually ignored or suppressed by other information. For example, the percept of one sound (e.g., voices of neighbors) could be suppressed by another (e.g., the sound of a car horn close to us). In this case, the latter input is dealt with preferentially in terms of physical (louder) and ecological (warning) priorities. As such, the suppressive mechanism in integration processes could also play an important role in the appropriate formation of our percepts. Many studies have demonstrated perceptual suppression effects in single modalities (e.g., vision^2^,^3^,^4^ and audition^5^,^6^). A neurophysiological study^7^ also reported that the visual perceptual masking effect occurred due to direct interactions of neural responses. This study demonstrated that, when the percept of a target stimulus was suppressed by another masker stimulus presented immediately before or after the target, neural responses related to the onset or offset of the target were also suppressed by the masker.

Thus far, studies on crossmodal interactions have mainly reported on the additive/facilitatory aspects of the interactions. For example, auditory stimuli were reported to enhance the perceived intensity of a visual stimulus^8^. Consistent with this behavioral evidence, the pooling of neural signals to multimodal stimuli have been demonstrated to induce facilitatory interactions of these signals in the superior colliculus (SC) of cats^9^,^10^. Based on findings like these, the importance of crossmodal interactions has been regarded as an additive/facilitatory integration of multimodal stimuli^11^,^12^. This process was assumed to occur not only at some higher sensory association areas^13^ but also at primary sensory cortices, which have been assumed to be single sensory specific areas^14^.

It should be noted, however, that some studies have also reported inhibitory/suppressive neural interactions for crossmodal inputs. For example, the pooling of neural signals to multimodal stimuli also elicited inhibitory responses in the SC of cats^9^,^10^. Moreover, a noise burst was recently reported to directly inhibit the neural responses to a light in the primary visual cortex of mice, resulting in the suppression of their motor responses induced by visual stimulation^15^. A human brain imaging study also showed that auditory or visual stimuli deactivated a portion of the visual or auditory related cortices, respectively^16^.

These findings clearly suggest the existence of suppressive/inhibitory processes for crossmodal interactions in the brain due to direct interactions of neural responses. However, to the best of our knowledge, only one study has provided psychophysical evidence showing the crossmodal (visuo-tactile) direct perceptual suppressive effect in human behavioral performance^17^. In particular, no direct evidence regarding audio-visual perceptual suppressive effect has been reported so far, although neural inhibitory interactions have been reported mainly across auditory and visual modalities^9^,^10^,^15^,^16^.

The current study investigated whether sound could induce a suppressive effect on visual perception. In our experiments, we presented white noise bursts and a visual target stimulus (Gabor patches) (Fig. 1). We adopted a visual orientation discrimination task to isolate a perceptual effect instead^17^, since other types of tasks, such as a simple detection task, may involve a response/decisional bias (e.g., the presentation of sound induces frequent visual present responses/judgments). We found that sounds presented through headphones degraded visual orientation discrimination performance. This auditory suppression effect on visual orientation discrimination frequently occurred when these inputs were presented in a spatially and temporally consistent manner. These results indicate that the perceptual suppression effect could occur across auditory and visual modalities.

## Results

### Auditory suppression effect on visual discrimination (Exp. 1)

**Figure 1:**
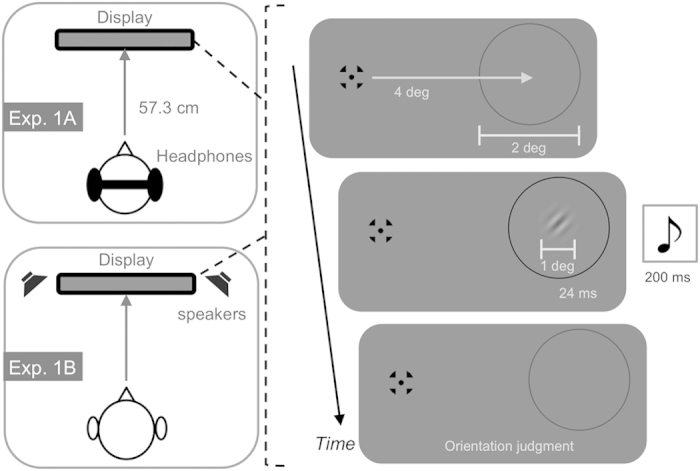
Schematic illustrations of the experimental situation in Exp. 1. After the presentation of a fixation point (0.6 × 0.6 deg) and a gray ring (2 deg in diameter, 0.05 deg in width) for 1200−1500 ms (randomly assigned in each trial), a visual target (1 deg) tilting either left or right was presented at the center of the ring for 24 ms. The ring color was also changed from gray to black as the cue for the target onset. White noise bursts were presented through headphones (Exp. 1A) or loud speakers (Exp. 1B) for 200 ms. Participants were asked to judge the target’s orientation.

We presented Gabor patches (1 × 1 deg, 4 cycle/deg, σ = 0.17 deg, 180 deg of phase angle) as target stimuli at the right side of the visual field (4 deg apart from the center) against a gray background (29.98 cd/m^2^) for 24 ms. A gray ring (2 deg) was also presented around the target and its luminance was changed (from 15.03 to 0.1 cd/m^2^) to cue the onset of the target. In the first session, we ran two adaptive staircase sequences (ascending and descending series) to determine a visual contrast threshold value (Weber contrast) for the 70.7% orientation discrimination performance, without sound, for each participant. We asked the participants to judge whether the orientation of the target was perceived as tilting left (−45 deg) or right (+45 deg) (Fig. 1). The target’s contrast changed in response to the participant’s judgment (correct/incorrect) in each trial. The contrast values of the last 10 response reversal points in the two sequences were averaged to estimate the threshold.

In the second session, targets with the estimated contrast threshold value were presented for each participant. Concurrently, white noise bursts (200 ms with 5 ms of rise and fall) were delivered with 45, 55, or 65 dBA of sound pressure levels (SPL). We presented these sounds through headphones to eight participants (Exp. 1A). The participants were asked to judge the orientation of the target while ignoring the sounds. Trials without any sound were also introduced as a baseline (no-sound condition).

**Figure 2:**
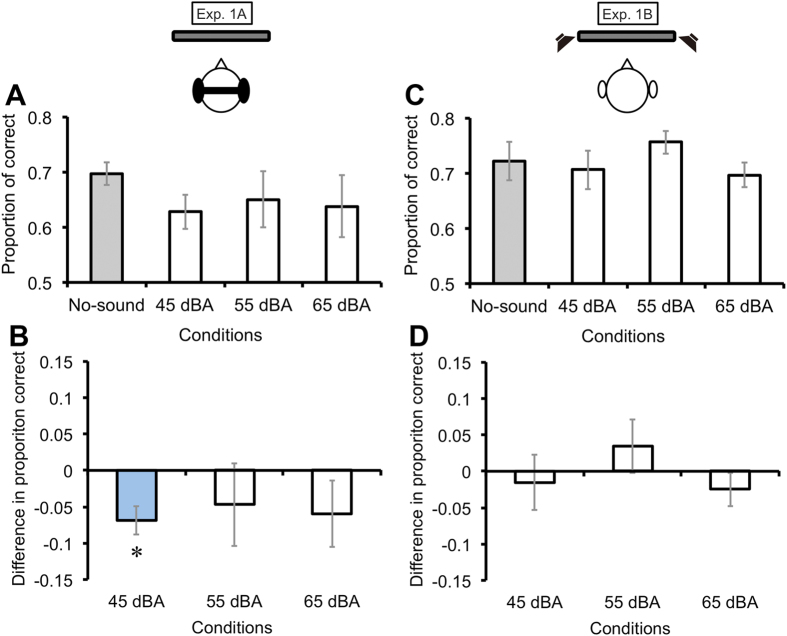
Results from Exp. 1. From the proportion of correct responses (**A**, **C**), we calculated the differences in proportion correct by subtracting the proportion correct in the no-sound condition from that in each sound condition (**B**, **D**). The visual orientation discrimination performance was degraded only when the 45-dBA SPL sound was presented through the headphones. Error bars denote the standard error of the mean (N = 8). An asterisk indicates the sound condition significantly different from the baseline (the no-sound condition) (*p* < .05).

From the proportions of correct responses in each condition (Fig. 2A), we calculated the differences in proportion correct by subtracting the proportion of the no-sound condition from that of each sound condition (Fig. 2B). To determine which sound conditions had a significant effect on visual orientation discrimination in comparison to the baseline, we conducted pairwise t tests (*p* < .05) with Bonferroni corrections between the no-sound condition and each sound condition. The results revealed that the proportion of correct responses in the 45-dBA SPL sound condition was significantly lower than that in the no-sound condition (*ts*(7) = −3.56, −0.82, and −1.30 in the 45-, 55-, and 65-dBA SPL sound conditions, respectively). We also calculated effect sizes for each sound condition against the no-sound condition^18^,^19^. The 45-dBA SPL sound condition had a large effect size (*d*_D_ = −1.26), whereas the 55-dBA SPL and 65-dBA SPL sound conditions had over small (*d*_D_ = −0.29) and below medium (*d*_D_ = −0.46) levels, respectively. These results indicate that sound with a particular SPL can suppress the perception of visual stimuli.

The literature regarding crossmodal interactions has reported that the magnitude of crossmodal interactions became stronger when the stimuli in a different modality were presented in a spatially co-localized manner^11^. In Exp. 1B, sounds were presented to an additional eight participants through speakers set in a nearby display (Fig. 1). We did not find significant effects for any sound condition in comparison to baseline (*ts*(7) = −0.41, 0.93, −1.08 in the 45-, 55-, and 65-dBA SPL sound conditions, respectively) (Fig. 2C,D), and the effect sizes for each sound condition against baseline were lower than small or medium level (*d*_D_ = −0.15, 0.33, and -0.38 in the 45-, 55-, and 65-dBA SPL sound conditions, respectively). These results suggest that the auditory suppression effect could occur specifically when the sounds were produced close to the participants’ heads.

### Spatial congruency aspect of the auditory suppression effect on visual discrimination (Exp. 2)

**Figure 3:**
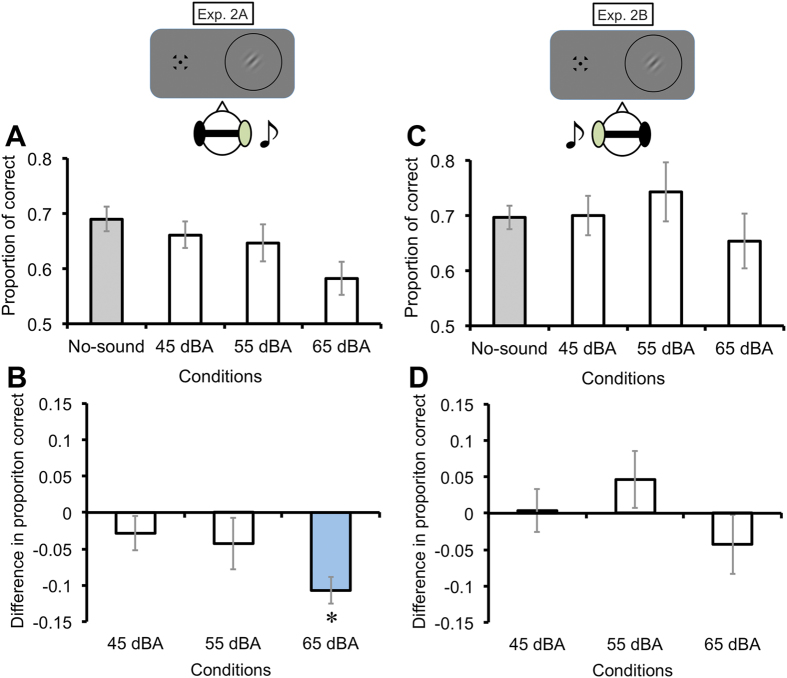
Effects of spatial congruency (Exp. 2). The visual target was presented at the right side of the display, whereas the sounds were provided to the participant’s right (Exp. 2A: congruent) or left ear (Exp. 2B: incongruent) through headphones. From the proportion correct (**A**, **C**), the differences in proportion correct (**B**, **D**) were calculated by subtracting the proportion correct in the no-sound condition from each sound condition. Visual orientation discrimination performance was degraded only when the 65-dBA SPL sound was presented spatially congruent with the visual target. Error bars denote the standard error of the mean (N = 7). An asterisk indicates the sound condition significantly different from the baseline (the no-sound condition) (*p* < .05).

We also investigated spatial aspects of the auditory suppression effect on visual perception. In Exp. 2A, the sounds were delivered from the right side of the headphones for seven participants, and the visual target stimulus was presented at the right side of the visual field. Regarding the differences in the proportion of correct responses from each sound condition in comparison to the no-sound condition (Fig. 3A,B), pairwise t tests (*p* < .05) with Bonferroni corrections revealed that the proportion of correct responses in the 65-dBA SPL sound condition was significantly lower than that of the no-sound condition (*ts*(6) = −1.22, −1.22, and −5.74 in the 45-, 55-, and 65-dBA SPL sound conditions, respectively). The 65-dBA SPL sound condition also had a large effect size against baseline (*d*_D_ = −2.17), whereas the effect sizes for the 45-dBA and 55-dBA SPL sound conditions were below medium level (*d*_D_ = −0.46, −0.46). On the contrary, when the sounds were presented from the left side of the headphones for seven additional participants (Exp. 2B), we did not find any significant auditory effect on visual discrimination performance (*ts*(6) = 0.12, 1.19, and −1.05 in the 45-, 55-, and 65-dBA SPL sound conditions, respectively) (Fig. 3C,D). The effect size for each sound condition was also lower than small or medium level (*d*_D_ = −0.05, 0.45, and –0.40 in the 45-, 55-, and 65-dBA SPL conditions, respectively). These results indicate that the auditory suppression effect on visual perception became dominant when both stimuli were presented in an ipsilateral, spatially congruent manner.

### Temporal congruency aspect of the auditory suppression effect on visual discrimination (Exp. 3)

**Figure 4:**
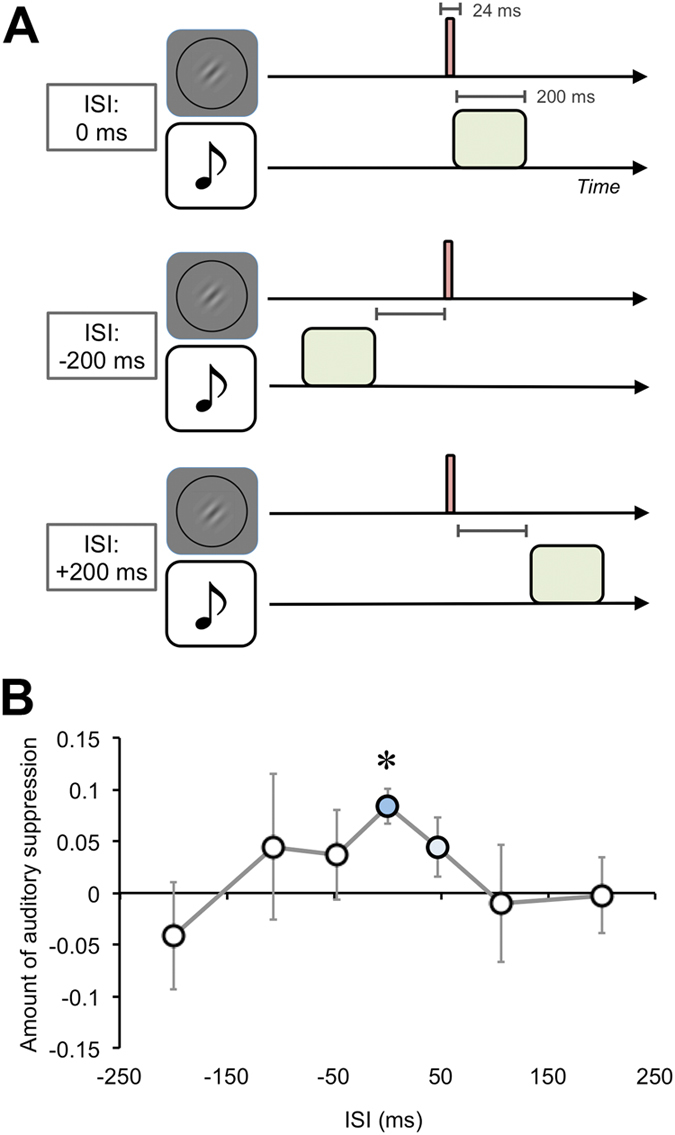
Effects of temporal congruency (Exp. 3). (**A**) Schematic illustrations of the inter-stimulus interval (ISI) between the visual and auditory stimuli. ISIs of 0, ±47, ±106, and ±200 ms (minus sign indicates auditory stimulus first and plus sign indicates visual stimulus first) were assigned. (**B**) Results. The magnitude of the auditory suppression effect in each ISI condition was calculated based on proportions of correct responses for visual discrimination. A significant auditory suppression effect occurred in the 0-ms ISI condition. There was also a medium effect size in the + 50 -ms ISI condition. Error bars denote the standard error of the mean (N = 8). An asterisk indicates the condition significantly different from the baseline (the no-sound condition) (*p* < .05).

We further investigated the temporal aspects of the auditory suppression effect on visual perception. The duration of the auditory (200 ms) and visual (24 ms) stimuli were different, and the auditory suppression effect could occur when these stimuli overlapped temporally. Thus, we manipulated the inter-stimulus interval (ISI) between the auditory and visual stimuli to be 0, ±47, ±106, and ±200 ms (minus sign indicates auditory stimulus first and plus sign indicates visual stimulus first) for eight participants (Fig. 4A). The auditory and visual stimuli were presented as spatially congruent so that the 65-dBA SPL sounds were presented from the right side of the headphones and the visual target stimuli were presented at the right side of the display. The no-sound condition was also introduced as a baseline.

We estimated the magnitude of the auditory suppression effect by subtracting the proportions of correct responses in each ISI condition from those in the no-sound condition (Fig. 4B). Larger values indicate a greater suppression effect. The pairwise t test (*p* < .05) with Bonferroni corrections between each ISI condition and the no-sound condition indicated a significant difference between the 0-ms ISI condition and the baseline (*ts*(7) = −0.75, 0.60, 0.80, 4.63, 1.46, −0.17, and −0.06 in the −200, −106, −47, 0, +47, +106, and +200 ms conditions, respectively). We also calculated effect sizes for each ISI condition against baseline. The effect sizes of the 0-ms and +47-ms ISI conditions were large or medium (*d*_D_ = 1.64 and 0.52, respectively), whereas those in the other conditions were over or below small level (*d*_D_ = −0.26, 0.21, 0.28, −0.06, and -0.02 in −200 -, −106 -, −47 -, +106 -, and +200 -ms ISI conditions, respectively). These results indicate that the auditory suppression effect on visual target discrimination predominantly occurs when these stimuli are presented in a temporally congruent manner.

## Discussion

The current study investigated whether sound could induce a suppressive effect on visual perception. We found that white noise bursts presented through headphones degraded visual orientation discrimination performance. This auditory suppression effect was not observed when the sounds were presented through speakers close to the visual display. We also revealed that the suppression effect occurred when the sound and visual target stimuli were presented in an ipsilateral, spatially congruent manner. Moreover, the auditory suppression effect predominantly occurred when the sound and visual target stimulus were presented in a temporally congruent manner. These results indicate that sound can induce a suppression effect on visual perception, particularly when the stimuli correspond spatially and temporally.

Some studies have reported crossmodal attentional effects. For example, a stimulus in one modality could capture the participants’ attention to the stimulus locations from another modality^20^. Moreover, it was reported that tactile stimulation increased the detection threshold for sounds^21^. This effect was assumed to occur based on attentional capture from a sound to a tactile stimulus^22^. In the current study, the auditory suppression effect on visual orientation discrimination performance did not occur when the sounds were presented close to the visual target display (via speakers) but occurred when the sounds were presented away from the display (via headphones). Thus, one might assume that the auditory suppression effect would be based on the crossmodal attentional capture effect: the presentation of the sounds close to the head diverts attention from the visual target on the display. However, these crossmodal attentional effects could not fully explain our current findings. The auditory suppression effect clearly appeared when the sounds from the headphones and the visual targets were presented at an ipsilateral position, but not a contralateral one, although we could expect that both would have similar attentional spatial capture effects or even that the latter situation would have a stronger effect. We also showed that the auditory suppression effect on visual perception was dominant when the auditory stimuli were presented concurrently (i.e., 0-ms ISI) or subsequently (i.e., +47-ms ISI) to the visual stimuli. These temporal characteristics could be incompatible with the crossmodal attentional capture effect because the effect became dominant when the attentional cueing stimuli preceded the other one^23^,^24^. These spatial and temporal congruency aspects could also disprove the possible involvement of response/decisional biases, in addition to the fact that we introduced the indirect perceptual task (visual orientation discrimination) rather than a simple detection task. Therefore, we could conclude that the auditory suppression effect occurs in perceptual processes.

Thus far, there has been only one human study to provide direct behavioral evidence of a crossmodal perceptual suppressive effect^17^. This study showed that tactile stimulation (vibration) applied to an observers’ index finger suppresses visual orientation discrimination performance. This tactile suppression effect occurred especially when the tactile and visual stimuli were presented in a spatially congruent manner (i.e., the tactile stimulus to the index finger and the visual target on the display were located on the same side). Moreover, the effect became dominant when these stimuli were presented within a 0- to 50-ms (tactile stimulus was presented subsequent to the visual target) temporal range. Since the auditory suppression effect on visual perception reported here has very similar phenomenal aspects, we could assume that these crossmodal perceptual suppression effects share common underlying mechanisms.

It could also be noteworthy that the auditory suppression effect did not occur when the sounds were presented through loud speakers near the display, but did occur when they were presented through headphones close to the participants’ heads, although the former case could be predicted to have a stronger effect based on the spatial co-localization rule of crossmodal interactions^11^. This may simply suggest that sounds presented directly to the participants’ heads would be optimal for the current phenomenon counter to the spatial co-localization rule^25^. Notably, a greater auditory suppression effect was observed when the laterality of the sounds could be clearly introduced in a monaural presentation, as compared to when the sounds were presented as localized at center in a binaural presentation. We found that the 65-dBA SPL sounds induced 10% of the suppression effect in the monaural presentation (Exp. 2A), while 5% of the suppression effect was observed for the 45-dBA SPL sounds in the binaural presentation (Exp. 1A). The differences in the magnitude of the effect between the binaural and monaural presentations would indicate that the strength of the suppression effect increased when the spatial or hemispheric congruency is stressed by the monaural presentation. The differences in SPLs could simply be interpreted as a difference between optimal SPLs in the monaural and binaural presentations since no correspondence in SPL between these presentations could be assumed, considering that the amount of binaural summation for upper-threshold sounds would be 6–10 dBA at the utmost^26^. Similarly, the tactile suppression effect was reported to occur for tactile stimulation to the hand (index finger) but not to the forearm or chest^17^. We could assume that the crossmodal perceptual suppression effects also have some unique selectivity properties in each sensory pairing.

The current study demonstrates the suppressive/inhibitory perceptual effect of audition on vision. However, previous research on audio-visual interactions has mainly demonstrated the additive/facilitatory integration of multimodal stimuli^11^,^12^, even though the experimental frameworks were similar to that used in the current study. For example, researchers using detection tasks have consistently demonstrated auditory facilitatory effects on visual detection performance, and the involvement of one or more crossmodal effects, including response biases, attentional capturing to the target, temporal cueing to target onset, and perceptual enhancement, has been assumed^27^,^28^,^29^,^30^,^31^. Some researchers have also demonstrated auditory facilitatory effects on visual orientation discrimination performance; visual contrast thresholds for orientation discrimination task decreased by concurrently presented sounds^32^ and orientation discrimination sensitivity for the visual target increased by concurrent looming sounds^33^. These studies presented visual and auditory stimuli at same durations and the visual stimuli were presented as visible (contrast value for 82% orientation discrimination level^32^ or maximum contrast^33^), indicating that the perceptual intensities of the visual stimuli could be comparable to those of the auditory stimuli. Additionally, it was reported that sounds degraded visual orientation discrimination sensitivity^30^. In this study, two visual targets (Gabor patches) containing different orientation information (vertical and slightly tilted) were sequentially presented at different temporal intervals, and the participants were asked to indicate which interval contained the vertical target. A sound or no sound was presented concurrently with each target onset. The results showed that the discrimination threshold in orientation differences increased when the sounds were presented after the visual target presentation (30–60 ms of ISIs), but no such effects occurred when the onsets of the auditory and visual stimuli were nearly synchronized (0 ms of ISI). Since this previous study measured the threshold of orientation discrimination sensitivity (i.e., discriminability), rather than the contrast threshold (i.e., visibility), with the sequential presentation of each pair of the auditory and visual stimuli, the auditory stimuli may induce processes other than direct perceptual suppression of the visual target stimuli, such as confusion in discrimination/identification^30^ and/or enhancement of perceptual grouping^34^,^35^.

Neurophysiological studies have reported both facilitatory and inhibitory neural responses to audio-visual inputs in the SC of cats^9^,^10^, although the conditions under which these responses become dominant is still under debate. Interestingly, a human brain imaging study reported that the presentation of a single modality input (auditory or visual) deactivated the other sensory cortex (visual or auditory), while the presentation of upper-threshold multimodal (audio-visual) stimuli did not induce a similar inhibitory effect^17^. For upper-threshold multimodal stimuli, auditory facilitatory effects were observed in behavioral performances^32^,^33^. Notably, the current study demonstrates the auditory suppression effect on visual perception in a situation where the perceptual intensity of the visual target stimuli was lower than that of the auditory stimuli such that the visual target stimuli were presented near the threshold (contrast threshold value for 70.7% orientation discrimination) for a shorter duration (24 ms) against the upper-threshold auditory stimuli with a longer duration (200 ms). On the basis of this viewpoint, we could assume that one possible role of the crossmodal perceptual suppression effect would be to reduce the perceptual magnitude or inhibition of the percept of a relatively weaker or less salient modality input, leading to it being considered a “perceptual noise” in the background of the other stronger or more salient input. This idea is consistent with the traditional perceptual masking effect in a single modality^2^ and recent crossmodal studies (e.g., Refs. 36, 37). Furthermore, the sound-driven synaptic inhibition in the primary visual cortex of mice was reported to reset the temporal phase of synaptic oscillations in the visual cortex^15^. This finding would indicate that crossmodal perceptual masking also enables the brain to prepare for the establishment of robust and effective crossmodal integrations with subsequent reliable inputs by maintaining temporal consistency^38^. Detailed investigations will be necessary to elucidate the underlying mechanisms regarding how and why crossmodal perceptual suppression could occur in our brain, using neurophysiological and brain imaging techniques as well as psychophysical methods.

## Methods

### Ethics statement

The experimental procedures were approved by the local ethics committee of Rikkyo University, and were performed in accordance with the approved guidelines and the Declaration of Helsinki. Informed consent was obtained from each participant before conducting the experiments.

### Participants and apparatus

There were 38 participants in total (16, 14, and 8 participants in Exps. 1, 2, and 3, respectively). They had normal or corrected-to-normal vision and normal hearing. The participants were naïve to the purpose of the experiment. The visual stimuli were presented on a linearized CRT display (EIZO FlexScan T776, 19 inch) with a resolution of 1280 × 1024 pixels and a refresh rate of 85 Hz. The viewing distance was 57.3 cm. The auditory stimuli were generated digitally (22.05 kHz sampling frequency) and presented through an audio interface (Roland, EDIROL UA-25 EX) and headphones (Sennheiser HDA200) or loud speakers. A customized PC (Dell Precision T5500 workstation) and MATLAB (MathWorks, Inc.) with the Psychophysics Toolbox^39^,^40^ were used to control the experiment. A numeric keypad was used to record responses. We confirmed that the onset of the visual and auditory stimuli was synchronized using a digital oscilloscope (OWON, PDS5022TFT). The observers were instructed to place their heads on a chin rest. All experiments were conducted in a dark room.

### Stimuli

For visual stimuli, a fixation point consisting of a bull’s-eye and crosshair (0.6 × 0.6 deg; 0.1 cd/m^2^)^41^ and a gray ring (2 deg in diameter; 0.05 deg in width; 15.03 cd/m^2^) was presented on a gray background (29.96 cd/m^2^). The ring was presented at a position to the right of the fixation point at 4 deg of horizontal distance, and its color was changed from gray to black (0.1 cd/m^2^) during target presentations. We also presented a Gabor patch (1 × 1 deg, 4 cycle/deg, σ = 0.17 deg, 180 deg of phase angle) as the target for 24 ms at the center of the ring. The stripes of the target stimulus tilted either left (−45 deg) or right (+45 deg). For the auditory stimulus, white noise bursts were presented for 200 ms with a 5 ms cosine ramp at the onset and offset. Whereas the sounds were presented through the loud speakers in Exp. 1B, they were provided through the headphones in the remaining experiments. The SPLs of the sounds were 45, 55, and 65 dBA in Exps. 1 and 2, and 55 and 65 dBA in Exp. 3.

### Procedure

At the beginning of each experiment, we ran two adaptive, ascending and descending staircase sequences with a “2-up, 1-down” rule to determine a visual contrast threshold value for the 70.7% orientation discrimination for each participant (threshold estimation session). After presentation of the fixation point and the gray ring for 1200–1500 ms (randomly assigned in each trial), the visual target was presented. The ring color also changed from gray to black as the cue of target onset. No sounds were presented during the session, but the participants wore the headphones in all the experiments other than Exp. 1B. We asked the participants to judge whether the orientation of the target was perceived as tilting left or right. The target’s orientation was randomly assigned in each trial. The target’s contrast (Weber contrast) changed in response to the participant’s judgment (correct/incorrect) in each trial. While the descending series started from 40% of the target contrast, the ascending series began from 5% contrast. Each sequence was terminated when 15 response reversal points were obtained. The contrast values of the last 10 points of the two sequences were averaged to estimate each participant’s threshold, and the value was adopted as the target contrast in the subsequent main session. The averaged contrast threshold values (SD) were 6.73% (1.63%), 7.37% (2.82%), 9.16% (4.30%), 8.75% (2.00%), and 8.99% (2.40%) in Exps. 1A, 1B, 2A, 2B, and 3, respectively.

In the main session of Exp. 1, the sounds were presented with a 45-, 55-, or 65-dBA SPL concurrent with the onset of the visual target. We asked the participants to judge the orientation of the target while ignoring the sounds. The situation without the sounds was also included as baseline (no-sound condition). Except for these differences, the procedures were identical to those in the threshold estimation session. The sounds were presented through the headphones (Exp. 1A) or loud speakers (Exp. 1B). The participants were different in each experiment. Each experiment consisted of 160 trials: Auditory conditions (4) × Repetitions (40). The order of these conditions was randomly assigned in each trial and counterbalanced among the participants.

In Exp. 2, we presented the auditory stimulus either from the right ear (Exp. 2A) or the left ear (Exp. 2B) of the participants through the headphones. The visual target was presented at the right side of the display so that the former situation was regarded as spatially congruent, and the latter as incongruent. The participants were different in each experiment. Except for these differences, the procedure was identical to that in Exp. 1.

In Exp. 3, we manipulated the ISI between the visual targets and the 65-dBA SPL of the auditory stimulus: 0, ±47, ±106, and ±200 ms (minus sign indicates auditory stimulus first and plus sign indicates visual stimulus first). The auditory stimulus was presented to the right ear of the participants through the headphones. The visual target was presented at the right side of the display. In addition to the baseline condition with no sound, we presented the 55-dBA SPL sounds as filler to maintain the saliency of the 65-dBA SPL sounds. This experiment consisted of 280 trials: ISIs (7) × Auditory conditions (2: no-sound and 65-dBA SPL sounds) × Repetitions (16) + ISIs (7) × Filler (1: 55-dBA SPL sounds) × Repetitions (8). The order of these conditions was randomized in each trial and counterbalanced among the participants. Except for these differences, the procedure was identical to those in the other experiments.

## Additional Information

**How to cite this article**: Hidaka, S. and Ide, M. Sound can suppress visual perception. *Sci. Rep.* **5**, 10483; doi: 10.1038/srep10483 (2015).
